# Measuring depression in adolescence: Evaluation of a hierarchical factor model of the Children’s Depression Inventory and measurement invariance across boys and girls

**DOI:** 10.1371/journal.pone.0249943

**Published:** 2021-04-08

**Authors:** Martin Jelínek, Petr Květon, Iva Burešová, Helena Klimusová

**Affiliations:** 1 Faculty of Arts, Masaryk University, Brno, Czech Republic; 2 Faculty of Education, Masaryk University, Brno, Czech Republic; Guangzhou University, CHINA

## Abstract

**Background:**

One of the most widely used instruments to measure depression in childhood and adolescence is Kovacs’s Children’s Depression Inventory (CDI). Even though this particular measure sparked massive interest among researchers, there is no clear consensus about its factorial structure. It has been suggested that inconsistencies in findings can be partly ascribed to the cultural context. The aim of this study was a) to examine and verify the factor structure of CDI in the Czech population and b) to assess gender-related psychometric differences using the mean and covariance structure (MACS) approach and differential item functioning (DIF) analysis.

**Methods:**

The research sample consisted of 1,515 adolescents (ages 12 to 16 years, 53.7% female) from a non-clinical general population. Based on exploratory factor analysis (EFA) on a random subsample (N = 500), we proposed a model that was subsequently tested on the rest of the sample (N = 1,015) using confirmatory factor analysis (CFA). Following the MACS procedure, we assessed measurement invariance in boys and girls. The between-group comparison was further supplemented by a DIF analysis.

**Results:**

The proposed hierarchical four-factor model (General Symptoms, Negative Self-Concept, Inefficiency, and Social Anhedonia) with a second-order factor of depression fitted the data reasonably well (χ^2^ = 1281.355; df = 320; RMSEA = 0.054, CFI = 0.925). Regarding gender differences, we found no substantial signs of measurement invariance using the MACS approach. Boys and girls differed in first-order latent means (girls scored higher on General Symptoms with a standardized mean difference of 0.52 and on Negative Self-Concept with a standardized mean difference of 0.31). DIF analysis identified three items with differential functioning. However, the levels of differential functioning were only marginal (in two items) or marginal/moderate and the presence of DIF does not substantially influence scoring of CDI.

**Conclusion:**

In the general adolescent population in the Czech Republic, the CDI can be considered a reliable instrument for screening purposes in clinical settings and for use in research practice. Instead of the originally proposed five-factor model, we recommend using the newly established four-factor structure. The measure seems to show only marginal psychometric differences with respect to gender, and overall measurement invariance in boys and girls seems to be a tenable assumption.

## Introduction

Depression in adolescents is a global public health concern. The turbulence associated with physical, cognitive, emotional, and social development may play a principal role in the emergence of depressive symptoms in this developmental stage. While depressive symptoms, sadness, and negative emotions in general might, to some extent, be viewed as a natural aspect of the adolescent experience, they might also easily get out of hand and even result in the onset of clinical depression in later life [1]. In the scientific literature, the most commonly found explanation of depression is offered by the bio-psycho-social model, which is based on the idea that the causal background of depression consists in the interaction of biological, psychological, and social factors [2]. In this view, depression is a function of a broad range of biological, interpersonal, cognitive, behavioral, and socio-cultural influences, and none of the existing major theories of depression (i.e. psychoanalytic, behavioral, learned-helplessness, cognitive, stress, self-control, sociological, genetic, biochemical, etc. models) can, by itself, provide a satisfactory account of the etiology of depressive disorder or its various symptoms [3].

Depressive symptoms of different kinds can be found in approximately 25% of adolescents [4]; sometimes, however, they are erroneously identified as conduct disorders or somatic issues [5]. This happens despite the fact that adolescent symptoms of depression do not differ substantially from those found in adult depression–depressed adolescents, just like depressed adults, are unable to feel joy, suffer from low self-esteem, feelings of guilt, irritation, loneliness and social isolation, and experience protracted sad or even despondent moods [6]. Increased incidence of these symptoms in adolescence presents a risk factor not only for the development of adult depression, but also for undesirable high-risk behaviors, such as substance abuse [7], smoking and alcohol use [8], self-harm and suicidal behavior [9], and decreased school performance [10], among others. The presence of depressive symptoms at this developmental stage is therefore of high clinical significance especially in the prospective sense, i.e. because it represents a risk factor for maladaptive development with serious consequences for the individual’s mental health and quality of life [11].

Both in research practice and clinical settings, brief self-report questionnaires represent an efficient way for the purposes of screening depression in adolescence. Commonly utilized scales include e.g. Patient Health Questionnaire (both in 9-item and 2-item versions) [12], the 30-item Reynolds Adolescent Depression Scale [13], or the 33-item Mood and Feelings Questionnaire (MFQ) [14]. In the larger literature, the most widely used instrument for assessing youth depressive symptoms is the Children’s Depression Inventory [15], which originated as a downward extension of the Beck Depression Inventory [16].

### The Children’s Depression Inventory (CDI)

The appropriateness of psychological assessment tools in adolescence is determined by the unique aspects of its individual sub-stages. At the beginning of early adolescence, depression is still most commonly diagnosed with the help of behavioral indicators obtained through direct observation, play, or projective drawing tests. At later stages, as the ability to self-reflect gradually develops, it becomes increasingly possible to employ instruments intended for adults, i.e. scales and inventories. The most commonly used tool for the assessment of depressive symptoms in adolescence is the Children’s Depression Inventory (CDI) by Maria Kovacs [17], who derived the instrument from the Beck Depression Inventory [16]. Kovacs describes the inventory as a measure of the current level of depression, which can also be used to capture changes in depressive states. As a screening measure, the CDI cannot, by itself, be used to make a diagnosis of clinical depression, which would require a more complex individual assessment. After its publication, the CDI relatively quickly became widely used among both practitioners and researchers, with 23 different language adaptations by 2003 [18], which might be partly thanks to its fast and simple administration (15 minutes or less), and the fact that the measure is suitable for a wide age range of 7 to 17 years. While primary assessment is based on the interpretation of the total score, it is also possible to interpret individual scores of the five subscales (Negative Mood, Interpersonal Problems, Anhedonia, Negative Self-Esteem, and Ineffectiveness) into which the 27 CDI items have been formally divided [17]. This originally proposed subdivision of the scale was also adopted by the author of the Czech adaptation [19]. However, the internal structure of the CDI has become a widely debated issue among researchers, with dozens of studies published in the years following the introduction of the measure.

### Variations in the factorial structure of the CDI

As early as 1983, Hodges et al. [20] identified four factors in the CDI on a sample of child inpatients at a mental hospital. These factors represented cognitive, motivational, social-inclusion, and somatic components of depression. In a non-clinical sample, however, the authors only detected two factors, one of which covered general, non-differentiated aspects of depression; the other reflected aspects of noncompliant behavior. In contrast, Saylor et al. [21] extracted as many as seven factors in child and adolescent inpatients and eight factors in healthy children; dominant factors, mainly reflecting feelings of inferiority and low self-esteem, did not differ much across the two samples. Craighead, Smucker, Craighead and Ilardi [22] pointed out that the number of factors extracted with the CDI might vary depending on the age of respondents. These authors identified five factors in a sample of children (Externalizing, Dysphoria, Self-Deprecation, School Problems, and Social Problems) and one additional factor (Biological Dysregulation) in an adolescent sample. Thus, it can be said that while all of these findings strongly indicate that the CDI is not a unidimensional measure, they also suggest that the number and nature of the individual factors depends on the characteristics of the research sample.

A comprehensive meta-analysis of 24 studies with 35 samples [23] demonstrated that there is little empirical justification for the originally proposed CDI subscores. The authors performed a factor analysis with primary pattern matrices obtained from psychometric studies that employed English versions of the CDI. While the number of identified factors corresponded to the original five-dimensional model, these factors were completely different from those proposed by Kovacs in terms of content. The only factor that clearly overlapped with the original model was Negative Self-Concept; the other factors, labelled as Somatic Concerns, Externalizing, Lack of Personal and Social Interest, and Dysphoric Mood, were not analogical to any of Kovacs’s subscores. The meta-analysis also revealed that translation into other languages might act as a significant moderator of the scale structure. In contrast to the English version, the model that showed the best fit with data obtained with non-English adaptations was a four-factor model with factors Sadness and Somatic Concerns, Externalizing, Lack of Personal and Social Interest and Loneliness, and Negative Self-Concept. The authors of the meta-analysis conclude that the English and non-English versions of CDI show concept dissimilarity and suggest that it can be caused by interaction between cultural differences and CDI characteristics. Further evidence of the culture-dependent structure of the CDI is provided by more recent studies. Researchers have, for example, obtained a simple unidimensional structure with Native American and Native Alaskan adolescents [15], a two-factor structure with Nigerian adolescents [24], and even Kovacs’s originally suggested five-factor model with Australian adolescent respondents [25]. Given the fact that the three above mentioned studies performed on English speaking samples resulted in three highly different suggestions of underlying factor structure of CDI, it is important to acknowledge that significant differences in the conceptualization of depressive symptoms may exist between cultures, despite their shared language [26].

The diversity in the factorial structure results can be ascribed both to methodological issues and differences in the manifestations of depressive symptoms across samples. Many early studies suffered from significant methodological limitations, such as small sample sizes (as pointed out by Lee et al. [27]) or inappropriate (orthogonal) rotation in factor analysis [28]. On the other hand, some authors identified substantial sample-specific instability even across methodologically sound studies. This instability was manifested mainly in the non-core factors (beyond primary symptoms of depression, resembling Kovacs’s factors of Negative Mood, Interpersonal Problems, and Negative Self-Esteem). Despite the structural differences found, the identified factors were, in most cases, highly correlated. Therefore, Lee et al. [27] suggest that a single higher-order factor may best represent the structure of CDI. This hierarchical perspective is further supported by Craighead et al. [29], who reported that a global CDI score yielded comparable accuracy to the factorial scoring approach in discriminating depressed and non-depressed adolescents.

### Gender differences in the CDI

One of the most consistent findings in depression research concerns gender differences, with women experiencing depressive symptoms much more frequently than men [30]. From a developmental perspective, gender differences in depression and depressive symptoms emerge in middle adolescence [31, 32]. A question that remains unanswered, however, is whether these differences correspond to the actual experience or are simply a reflection of different ways that boys and girls perceive and respond to items in a particular instrument. Van Beek et al. [33] addressed this question in a large-scale study involving more than 4,000 schoolchildren and adolescent students and revealed significant measurement bias in the CDI, with multiple items showing differential item functioning with respect to age and gender. The general conclusion drawn by the authors was that the CDI overestimated depression in boys and girls in late childhood and underestimated depression in boys in middle adolescence. One possible explanation for this observation is that in boys depression might not always manifest itself through the standard depressive symptoms only but may also be accompanied by conduct disorders or substance abuse [34], neither of which is included in the CDI. Another study, specifically focused on potential measurement bias with respect to gender [35], found no evidence for bias; however, this might have been due to the young age of the participants (12 years or less).

### Aims

Given the inconsistent findings regarding the structure of the CDI, the first aim of our study was to specify and verify an appropriate hierarchical factor model in Czech population. The second aim was to explore gender differences in the instrument’s structure. We used the means and covariance structure (MACS) approach to examine measurement invariance for the proposed model across boys and girls and to compare the two groups in relevant first-order and second-order latent mean scores. We also used differential item functioning (DIF) analysis based on an item response theory (IRT) framework to further supplement the results from the factor-analytic approach, because it provides a way to empirically derive item bias parameters [36], and also can serve as supporting evidence for the stability of the results [37].

## Methods

### Sample and procedure

The data were obtained in school settings, using a paper/pencil questionnaire. The administration took place over one lesson. Originally, we collected data from 1,563 respondents from various basic education stage 2 schools in the Czech Republic. Cases with missing essential demographic variables (gender, age, or family completeness) and cases with more than three missing CDI item responses were removed, which reduced the sample to 1,515 cases (the demographic characteristics of the final sample are summarized in Table 1). The highest single-item lack in the resulting sample did not exceed 2.5%. Missing CDI responses were imputed using the expectation maximization algorithm in IBM SPSS v24.

**Table 1:** Sample characteristics.

Gender	*Male*	*Female*				
	46.3%	53.7%				
Age distribution	*12 y*.	*13 y*.	*14 y*.	*15 y*.	*16 y*.	
	3.3%	29.1%	36.5%	26.4%	4.7%	
Municipality size	*0–999*	*1*,*000–4*,*999*	*5*,*000–9*,*999*	*10*,*000–49*,*999*	*50*,*000–99*,*999*	*100*,*000+*
	3.8%	5.3%	20.5%	19.8%	19.7%	30.9%
Family completeness	*Complete*	*Incomplete*				
	69.0%	31.0%				

Note. Family completeness = the participant lives in a complete family, i.e. with both biological parents.

For analytical purposes, the sample was randomly divided into two subsets. One-third of the sample (N_S1_ = 500) was used for exploratory factor analysis (EFA), and the rest (N_S2_ = 1015) was used for confirmatory factor analysis (CFA) and measurement invariance (MI) testing. These two subsets were comparable in essential demographic characteristics: gender (S1: 52.8% females, S2: 54.1% females), age (S1: m = 13.98, sd = 0.93; S2: m = 14.01, sd = 0.94), and family completeness (S1: 69.4%; S2: 68.9%).

### Ethics

Approval for the project was obtained from the Research Ethics Committee of Masaryk University. Written informed consent was obtained from all participants and their legal representatives (parents or guardians).

### Measures

The Children’s Depression Inventory [17] is a self-rating scale for children and adolescents between 7 and 17 years of age. It consists of 27 symptom-oriented items with 3-point verbally anchored rating scales scored 0 to 2 with respect to the salience of depressive symptoms. Originally, Kovacs proposed a hierarchical model with a global depression scale and five formally differentiated (according to the area of symptom manifestation) subscales (see Table 2). Polychoric correlation-based McDonald’s ω of the full scale was 0.933 in the whole sample. The mean score in our sample was 11.99 (SD = 7.86).

**Table 2:** Descriptive statistics of the individual CDI items.

item # (symptom)	Original Kovacs’s subscale	Distribution of scores (% total)			Distribution of scores (% boys / girls)			*m* (total)	*m* (b / g)	r
		0	1	2	0	1	2			
1 (sadness)	A	71.7	25.5	2.8	84.2 / 60.9	14.1 / 35.3	1.7 / 3.8	0.31	0.43 / 0.18	0.725
2 (pessimism)	E	24.3	68.0	7.7	31.1 / 18.5	62.1 / 73.1	6.8 / 8.5	0.83	0.90 / 0.76	0.611
3 (self-criticism)	C	59.7	37.7	2.6	65.5 / 54.7	32.5 / 42.2	2.0 / 3.1	0.43	0.48 / 0.36	0.579
4 (anhedonia)	D	52.5	44.2	3.4	56.8 / 48.7	40.7 / 47.1	2.4 / 4.2	0.51	0.55 / 0.46	0.535
5 (misbehavior)	B	73.2	23.9	2.9	81.2 / 66.3	16.2 / 30.5	2.6 / 3.2	0.30	0.37 / 0.21	0.648
6 (pessimistic worry)	A	55.8	35.0	9.2	67.0 / 46.1	25.1 / 43.5	8.0 / 10.3	0.53	0.64 / 0.41	0.495
7 (self-hate)	E	77.1	19.9	3.0	86.2 / 69.2	11.3 / 27.3	2.6 / 3.4	0.26	0.34 / 0.16	0.712
8 (self-blame)	A	64.0	27.9	8.1	71.1 / 57.9	22.8 / 32.3	6.1 / 9.7	0.44	0.52 / 0.35	0.579
9 (suicidal ideation)	E	77.2	20.9	1.9	84.3 / 71.0	13.7 / 27.2	2.0 / 1.8	0.25	0.31 / 0.18	0.610
10 (tearfulness)	A	74.6	19.7	5.7	88.9 / 62.2	9.0 / 29.0	2.1 / 8.7	0.31	0.46 / 0.13	0.733
11 (irritability)	A	59.1	37.2	3.7	60.7 / 57.8	35.5 / 38.6	3.8 / 3.6	0.45	0.46 / 0.43	0.529
12 (antisocial feelings)	B	78.7	18.1	3.2	76.8 / 80.3	18.2 / 18.0	5.0 / 1.7	0.25	0.21 / 0.28	0.477
13 (indecisiveness)	A	47.1	46.1	6.9	51.4 / 43.3	42.0 / 49.6	6.6 / 7.1	0.60	0.64 / 0.55	0.563
14 (neg. self-image)	E	38.3	51.9	9.8	46.6 / 31.1	47.2 / 56.1	6.3 / 12.8	0.71	0.82 / 0.60	0.516
15 (school amotivation)	C	43.2	40.7	16.2	41.5 / 44.6	40.7 / 40.6	17.8 / 14.8	0.73	0.70 / 0.76	0.414
16 (sleep problems)	D	70.8	23.6	5.6	76.6 / 65.8	18.7 / 27.8	4.7 / 6.4	0.35	0.41 / 0.28	0.563
17 (fatigue)	D	48.6	42.0	9.4	57.3 / 41.2	34.6 / 48.3	8.1 / 10.5	0.61	0.69 / 0.51	0.474
18 (reduced appetite)	D	69.0	20.1	10.9	73.6 / 64.9	16.0 / 23.7	10.4 / 11.3	0.42	0.46 / 0.37	0.389
19 (somatic concerns)	D	46.0	46.1	7.9	59.1 / 34.7	36.0 / 54.7	4.8 / 10.6	0.62	0.76 / 0.46	0.559
20 (loneliness)	D	70.0	25.8	4.2	77.9 / 63.1	19.1 / 31.6	3.0 / 5.3	0.34	0.42 / 0.25	0.669
21 (boredom in school)	D	81.0	17.2	1.8	80.9 / 81.1	16.5 / 17.8	2.6 / 1.1	0.21	0.20 / 0.22	0.438
22 (few friends)	D	69.0	29.5	1.5	72.8 / 65.8	25.2 / 33.2	2.0 / 1.0	0.32	0.35 / 0.29	0.448
23 (academic decline)	C	53.2	40.3	6.5	55.1 / 51.5	38.3 / 41.9	6.6 / 6.5	0.53	0.55 / 0.51	0.555
24 (neg. peer comparison)	C	29.8	53.3	16.9	35.5 / 25.0	52.0 / 54.4	12.5 / 20.7	0.87	0.96 / 0.77	0.507
25 (feeling unloved)	E	72.4	24.9	2.7	71.9 / 72.8	24.4 / 25.3	3.7 / 1.8	0.30	0.29 / 0.32	0.524
26 (disobedience)	B	74.5	22.8	2.7	76.4 / 72.9	21.4 / 24.0	2.3 / 3.1	0.28	0.30 / 0.26	0.406
27 (fighting)	B	79.5	18.1	2.4	78.8 / 80.2	18.1 / 18.1	3.1 / 1.7	0.23	0.22 / 0.24	0.563

Note. 0 –no symptom, 1 –mild symptom, 2 –severe symptom; m–item mean; Kovacs’s original subscales: A–negative mood, B–interpersonal problems, C–ineffectiveness, D–anhedonia, E–negative self-esteem; r–corrected (item deleted) polychoric correlation between item and total score.

### Data analysis

Due to the relatively small number of verbally anchored response categories in the CDI, all data was treated as ordinal. Polychoric correlation matrices were used as input in both EFA and CFA. Where needed, item responses were reversed, so that higher values always indicated higher levels of depression.

To determine the number of factors to retain in EFA, we followed the recommendations provided by Courtney [38]. Five indices were taken into consideration that were obtained through the employment of minimum average partial (MAP), parallel analysis (PA), optimal coordinate (OC), acceleration factor (AF), and comparison data (CD) techniques. The subsequent exploratory factor analysis used maximum likelihood extraction method and direct oblimin rotation (delta = 0) to enable correlations between factors.

The model yielded by EFA was further evaluated through CFA with the WLSMV estimation method, which is considered as the best option for categorical or ordered data in the structural equation modelling context [39]. We used the comparative fit index (CFI) and the root mean squared error of approximation (RMSEA) to assess model fit with the following criteria suggested by Hu and Bentler [40]: good fit: RMSEA < .06 and CFI > 0.95; moderate/acceptable fit: RMSEA 0.6 to 0.8 and CFI 0.90 to 0.95. Estimation procedures were performed using the R package lavaan [41]. Finally, we tested measurement invariance for groups defined by gender. In this analysis, we employed the MACS approach to test the second-order factor structure as described by Byrne & Stewart [42]. This involved comparing a series of seven nested models. Due to the relatively large sample size, the χ^2^ difference was not used as an indicator of model misfit to avoid detection of trivial differences. Instead, we followed the guidelines proposed by Chen [43], suggesting a decrease of 0.01 or more in the value of CFI and an increase of 0.015 or more in RMSEA as an indication of lack of invariance. The first model (M1) was tested to assess configural invariance and served as a baseline model for comparison of all subsequent models. In this model, no parameters were constrained across groups. In the M2 model, item loadings were constrained to be equal across groups. In the M3 model, we added equality constraints on item thresholds; in the M4 model, item residuals were constrained. The three remaining analyses focused on the structural part of the hypothesized model, with constraints progressively placed on the second-order factor loadings (M5), first-order factor means (M6), and second-order factor mean (M7). The measurement invariance evaluation was further supplemented by between-groups latent means comparisons. When comparing first-order factor means, we used model M5 with first-order factor means set to zero in boys and freely estimated in girls and fixed the second-order latent means to zero in both groups. When comparing the second-order factor means, we used model M6 with the second-order factor mean set to zero in boys and freely estimated in girls. Standardized estimates of latent means in girls then represented the effect sizes, with appropriate statistical significance testing of group mean differences. In addressing the issue of statistical identification, first-order factor disturbances were constrained to equality in boys and girls in all models.

To examine potential differential item functioning (DIF), we used iterative hybrid ordinal logistic regression/IRT provided by the R package lordif [44]. The analysis employed the graded response model (GRM) for IRT trait estimation and McFadden’s pseudo R^2^ measure to identify items with uniform and non-uniform differential functioning across boys and girls in global depression. Items were flagged for DIF in case of values above 0.02 in the pseudo R^2^ change in the overall model (uniform plus non-uniform DIF). When considering the relevance of DIF, we used cut-off values of 0.035 (negligible vs. moderate DIF) and 0.070 (moderate vs. large DIF) proposed by Jodoin & Gierl [45], which are less conservative than the cut-offs (0.13 and 0.26, respectively) proposed by Zumbo [46]. To evaluate the impact of DIF, we quantified the relationship before and after DIF correction. More specifically, we computed the Pearson correlation coefficient between score estimates based on the overall item parameters and estimates based on the DIF-free and group-specific item parameters.

## Results

### Descriptive statistics of the CDI items

Table 2 provides a summary of the descriptive statistics (frequencies and means) of individual item scores together with item discrimination parameters. All items showed a high level of discrimination with respect to the total depression scale, ranging from 0.389 to 0.733. High skewness in the distribution of responses to all items provided further justification for our decision to treat data as ordinal in all subsequent analyses.

### Exploratory factor analysis

This analysis was performed on a random subsample (N = 500) taken out of the original dataset. First it was necessary to establish the most appropriate number of factors to be extracted. For this purpose, we used five different methods–MAP, PA, OC, AF, and CD. However, the results obtained with these methods were not fully consistent, and respectively proposed 2, 4, 4, 1, and 2 factors for extraction. It is well known that the AF index suffers from substantial under extraction [47], and we therefore considered only the two other solutions– 2 factors and 4 factors. Ultimately, we favored the four-factor structure, because we considered it important to preserve the content diversity of the original instrument and also because the PA index is the most recommended indicator of factor structure because of its unbiased nature [38]. Table 3 summarizes item factor loadings obtained through EFA after rotation, both from structure and pattern matrices.

**Table 3:** Results of EFA—The structure / pattern matrix and the factor correlation matrix.

Item #	F1	F2	F3	F4
10	**0.88 / 0.96**	-0.49 / 0.03	0.39 / 0.02	0.29 / -0.15
1	**0.85 / 0.84**	-0.51 / -0.01	0.29 / -0.13	0.49 / 0.14
20	**0.69 / 0.46**	-0.58 / -0.23	0.27 / -0.13	0.60 / 0.34
19	**0.68 / 0.69**	-0.38 / 0.04	0.22 / -0.11	0.39 / 0.12
5	**0.64 / 0.61**	-0.36 / 0.05	0.36 / 0.09	0.33 / 0.04
9	**0.64 / 0.46**	-0.56 / -0.26	0.35 / 0.05	0.34 / 0.02
16	**0.59 / 0.55**	-0.38 / -0.04	0.32 / 0.06	0.27 / -0.02
2	**0.59 / 0.35**	-0.51 / -0.20	0.43 / 0.16	0.39 / 0.11
6	**0.59 / 0.55**	-0.41 / -0.09	0.26 / -0.02	0.26 / -0.02
8	**0.59 / 0.39**	-0.50 / -0.20	0.54 / 0.34	0.20 / -0.16
3	**0.58 / 0.31**	-0.56 / -0.30	0.47 / 0.22	0.30 / -0.02
27	**0.48 / 0.27**	-0.36 / -0.05	0.44 / 0.26	0.36 / 0.14
18	**0.46 / 0.35**	-0.38 / -0.15	0.28 / 0.07	0.22 / -0.02
17	**0.42 / 0.29**	-0.23 / 0.09	0.37 / 0.22	0.35 / 0.18
7	0.66 / 0.17	**-0.99 / -0.94**	0.35 / -0.05	0.32 / -0.09
14	0.41 / -0.04	**-0.68 / -0.65**	0.29 / 0.03	0.35 / 0.12
25	0.47 / 0.12	**-0.48 / -0.24**	0.45 / 0.24	0.44 / 0.23
11	0.40 / 0.12	**-0.40 / -0.20**	0.37 / 0.19	0.36 / 0.18
15	0.16 / -0.13	-0.12 / 0.09	**0.74 / 0.86**	0.08 / -0.09
13	0.41 / 0.01	-0.38 / -0.08	**0.73 / 0.65**	0.38 / 0.14
23	0.38 / 0.03	-0.34 / -0.05	**0.68 / 0.62**	0.32 / 0.09
26	0.34 / 0.11	-0.24 / 0.03	**0.64 / 0.62**	0.17 / -0.06
24	0.35 / -0.02	-0.37 / -0.14	**0.60 / 0.52**	0.32 / 0.12
22	0.32 / -0.01	-0.20 / 0.11	0.20 / -0.02	**0.81 / 0.86**
21	0.38 / 0.03	-0.42 / -0.23	0.26 / 0.02	**0.56 / 0.45**
12	0.33 / 0.03	-0.33 / -0.14	0.27 / 0.09	**0.48 / 0.39**
4	0.43 / 0.24	-0.30 / 0.01	0.34 / 0.15	**0.44 / 0.28**
% EV	22.1	14.6	6.0	3.7
**Factor correlation matrix**				
	F1	F2	F3	**F4**
F2	-0.590			
F3	0.446	-0.376		
F4	0.459	-0.370	0.307	

Note. Factor loadings with highest values are in bold; % EV–percentage of explained variance before rotation.

It is evident that the items were distributed into the four factors unevenly. More than half of the items (14) loaded highest on the first factor, F1. These items mostly represented core depression symptoms, both emotional (e.g. sadness or tearfulness) and somatic (e.g. somatic concerns or sleep problems). Items in factor F2 generally reflected self-concept characteristics (e.g. self-hate, negative self-image, and feeling unloved). Since the F2 factor loadings have negative values (and all items were recoded to higher values indicate higher levels of depression), this factor needs to be interpreted as a positive self-concept in the EFA solution context. F2 is therefore negatively correlated with other factors, forming the expected pattern of relationships. The common denominator for items loading on factor F3 was performance issues (e.g. school amotivation or indecisiveness); for factor F4 it was anhedonia in social contexts (e.g. lack of friends or fun in school). Table 3 also shows that there were substantial correlations between factors, especially between F1 and the other three factors. The factor structure revealed through EFA was further tested using CFA on the remaining data from our dataset (N = 1,015).

### Confirmatory factor analysis (CFA)

**Fig 1:**
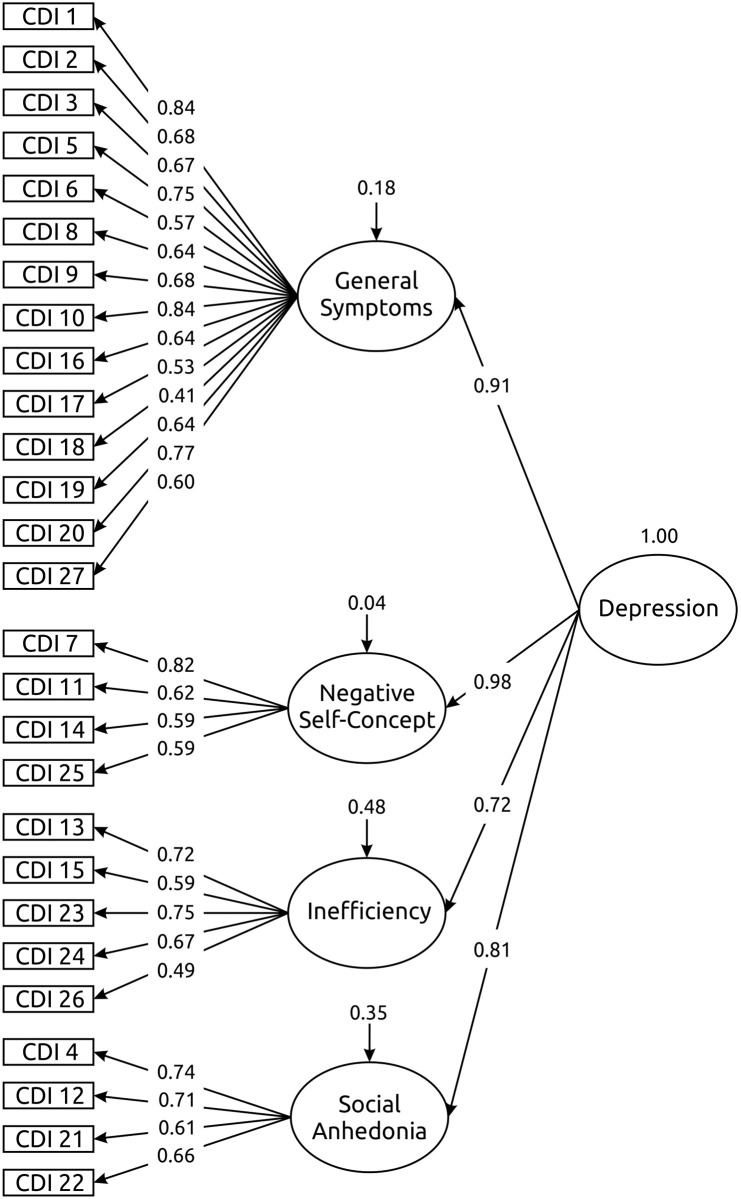
The second-order factor structure of the CDI. Standardized regression coefficients are stated; error terms (uncorrelated) are omitted for the sake of clarity.

Based on the factor loadings obtained through EFA, we proposed a four factor structure for the CDI with correlated factors. Indicators of the individual factors were selected on the basis of highest factor loading. The model as a whole fitted the data reasonably well (χ^2^ = 1261.721; df = 318; RMSEA = 0.054, 90% CI [0.051, 0.057]; CFI = 0.927), the values of fit indices suggested a good (RMSEA) or acceptable (CFI) fit. To further support our decision to favor the model with four first-order factors, we also tested the other potential structure of CDI with two first-order factors. All data fit indices for this model (χ^2^ = 1454.300; df = 323; RMSEA = 0.059, 90% CI [0.056, 0.062]; CFI = 0.912) were sufficiently high, though they were slightly worse in comparison to the four-factor model. Regarding the fact that the author of CDI suggested the existence of a hierarchical arrangement of the instrument [12], we proceeded by suggesting a second-order factor solution model with four first-order factors and one second-order factor. The data fit of this model was virtually on the same level (χ^2^ = 1281.355; df = 320; RMSEA = 0.054, 90% CI [0.051, 0.058]; CFI = 0.925) as the model with four correlated factors. In conclusion, the hierarchical factor model was preferred over the model with correlated factors; Fig 1 summarizes the standardized factor loadings for the model.

In the measurement part of the model, all items showed high loadings on appropriate factors (with the exception of items 18 and 26, all item loading values exceeded 0.5). In contrast to the EFA solution, items reflecting self-concept characteristics have positive factor loadings with the appropriate factor and thus we can label this factor as a Negative Self-Concept. The magnitudes of the second-order factor loadings reflected considerable mutual overlapping of the first-order factors, which could be sufficiently explained by the general single second-order depression factor.

### Measurement invariance and latent means comparison in boys and girls

To assess the invariance of the proposed second-order factor model across genders, we successively tested seven nested models. Models M1 to M4 were used to evaluate the measurement part of the model, while the structural part of the model was addressed in models M5 to M7. The results of all invariance tests are summarized in Table 4.

**Table 4:** Results of tests of the CDI measurement invariance across boys and girls.

Model	WLSMV χ^2^	df	RMSEA (90% CI)	CFI	constraints across groups (applied additionally)
M1	1429.909	644	0.049 (0.046–0.053)	0.934	no constraints
M2	1377.830	667	0.046 (0.042–0.049)	0.941	fixed first-order factor loadings
M3	1522.443	689	0.049 (0.046–0.052)	0.930	fixed thresholds
M4	1585.785	716	0.049 (0.046–0.052)	0.927	fixed residuals
M5	1612.859	719	0.050 (0.046–0.053)	0.925	fixed second order factor loadings
M6	1660.303	723	0.051 (0.047–0.054)	0.922	fixed first-order factor means
M7	1838.837	724	0.055 (0.052–0.058)	0.907	fixed second-order factor means

Note. WLSMV–weighted least square with means and variance adjusted; all χ^2^ were significant (p<0.001); RMSEA–root mean square error of approximation; CFI–comparative fit index.

When evaluating the measurement part of the model, the values in Table 4 clearly indicate that neither model M2 nor model M4 substantially deviated from model M1, which was used as the baseline model for all comparisons (differences in RMSEA indices did not exceed the 0.015 threshold, and CFI differences did not exceed the 0.01 threshold). When looking at the structural part of the model, it is apparent that fixation of the second-order factor loadings did not result in substantial deterioration in the model fit. In models M5 and M6, while the RMSEA indices did not show notable differences from model M1, the values of CFI indicated a decrease in model fit (ΔCFI = 0.012 and 0.017, respectively). In conclusion, the results suggest that the only source of misfit in the nested models were the equality constraints placed on factor means across the two gender groups. Therefore, we proceeded to test potential differences in the first-order and second-order latent factor means (see the Method section). We found that among the first-order factors the largest difference between boys and girls was in General Symptoms of depression, in which girls scored significantly higher than boys (standardized mean difference = 0.52, z = 6.423, p < 0.01). A smaller but still significant difference was found with the Negative Self-Concept factor, in which girls, again, scored higher than boys (standardized mean difference = 0.31, z = 3.477, p < 0.01). Boys and girls did not differ significantly in Social Anhedonia (standardized mean difference = 0.134, z = 1.735, p = 0.083) or Inefficiency (standardized mean difference = 0.05, z = 0.538, p = 0.591). Girls scored significantly higher than boys on the higher-order Depression factor (standardized mean difference = 0.41, z = 5.007, p < 0.01).

### Differential item functioning

A further elaboration of the gender-related measurement differences from a different perspective is offered by the DIF analysis. Potential DIF was evaluated at the level of the higher-order factor of global depression. Table 5 provides a summary of the results, with McFadden’s pseudo R^2^ indicating uniform, non-uniform, and overall DIF. We also computed GRM item parameters for all items.

**Table 5:** Results of DIF analysis and GRM item parameters (N = 1,515).

	DIF (McFadden’s R^2^)			GRM item parameters		
#	uniform	non-uniform	overall	a	b1	b2
1	0.014	0.000	0.014	2.42	0.71	2.37
2	0.000	0.000	0.000	1.66	-1.00	2.06
3	0.000	0.004	0.005	1.45	0.38	3.14
4	0.001	0.000	0.001	1.18	0.10	3.35
5	0.001	0.000	0.001	1.77	0.87	2.71
6	0.004	0.000	0.004	1.18	0.26	2.36
7	0.004	0.007	0.010	2.11	0.96	2.46
8	0.000	0.000	0.000	1.44	0.56	2.21
9	0.001	0.000	0.001	1.65	1.08	3.11
10	**0.038**	0.000	**0.038**	2.47 / 2.54	1.23 / 0.59	2.25 / 1.81
11	0.007	0.001	0.008	1.17	0.40	3.27
12	**0.025**	0.004	**0.029**	1.43 / 1.07	0.86 / 1.77	2.34 / 4.44
13	0.002	0.000	0.002	1.19	-0.12	2.65
14	0.002	0.000	0.003	1.16	-0.53	2.31
15	0.010	0.000	0.010	0.76	-0.40	2.40
16	0.000	0.000	0.000	1.36	0.87	2.62
17	0.001	0.000	0.001	1.02	-0.06	2.59
18	0.000	0.001	0.001	0.80	1.14	2.92
19	0.011	0.000	0.012	1.41	-0.16	2.23
20	0.000	0.000	0.000	1.95	0.70	2.33
21	0.009	0.000	0.009	0.98	1.74	4.54
22	0.001	0.010	0.011	0.94	1.00	4.92
23	0.007	0.000	0.007	1.16	0.14	2.75
24	0.001	0.000	0.001	1.07	-0.99	1.77
25	0.016	0.002	0.018	1.16	1.04	3.59
26	0.001	0.000	0.001	0.78	1.54	4.92
27	**0.020**	0.001	**0.021**	1.54 / 1.34	0.93 / 1.56	2.62 / 3.78

Note. R^2^ values exceeding the 0.020 threshold are in bold; a–item discrimination; b1, b2 –item threshold parameters; in case of DIF (R^2^ > 0.020), GRM item parameters were estimated separately (boys / girls).

We identified three items which could be considered to function differentially in boys and girls. DIF in all three items could be described as mostly uniform, i.e. the effect of groups does not substantially vary conditional on the level of depression and therefore it manifests itself mainly in the item threshold parameters b1 and b2. However, two of these items (27 and 12) showed only negligible levels of DIF, and the DIF level of item 10 was on the edge between negligible and moderate. When considering the same level of depression, boys have a greater probability of choosing a more symptomatic response with items 27 (fighting) and 12 (antisocial feelings); conversely, with item 10 (tearfulness), boys have a lower probability of symptomatic responses than girls. The impact of DIF items on the overall score of depression can be considered negligible as the correlation between the DIF-adjusted and the unadjusted persons’ scores was r = 1.000 (when rounding to three decimal places).

## Discussion

The CDI is the most widely used instrument for the assessment of symptoms of depression in the child and adolescent population. In clinical practice, the inventory has been in use for more than 40 years. Bearing in mind the diversity in depressive symptoms and the general multidimensionality of the depression syndrome, researchers have conducted a large number of psychometric studies directly focused on the factor structure of the CDI. Inconsistencies in results prompted [23] to conduct a psychometric meta-analysis, bringing together data from 24 independent studies. The meta-analysis yielded two important findings: first, the empirically derived structure did not correspond to Kovacs’s [17] original conceptualization; second, the structure showed cross-cultural differences.

A look at the individual published psychometric studies reveals considerable inconsistencies not only in the number, but also in the content and interpretation of the individual factors. For this reason, we decided to employ a combination of exploratory and confirmatory approaches in our study. EFA identified a four-factor solution as the most adequate one, with factors which we labelled as General Symptoms, Self-Concept, Inefficiency, and Social Anhedonia. This structure was subsequently tested further through CFA and turned out to be viable for our sample. Considering the heterogeneity of studies published on this topic, there is perhaps little use in discussing our results in the broad context of previous research. Instead, we would like to compare our findings with two principal reference criteria–the original five-factor model postulated by the author of the CDI [17], and the generalized four-factor structure obtained through the meta-analysis of psychometric studies of non-English adaptations of the instrument [23].

The most prominent feature of the structure identified in our study is the presence of a single dominant factor comprising more than half of all CDI items, which we labelled General Symptoms. This in itself indicates that the other identified factors were much more narrowly defined than those described by Kovacs or by Huang and Dong. In terms of content interpretation, one of our remaining factors corresponded to negative self-concept or low self-esteem, which is conceptually in line with both of the reference models. In all three factor structures, this factor is defined by symptoms of self-hate and negative self-image. In our structure, this factor also includes the feeling unloved item (as well as Kovacs’s structure) and the irritability item. In Kovacs’s interpretation, irritability belongs among symptoms of negative mood and in the meta-analytic study it came under Sadness and Somatic Concerns. Even though irritability is commonly counted among mood disturbances [48], adolescents’ poor self-concept has been related to many negative characteristics including irritability [49], which can explain assigning this item to the Negative Self-Concept factor in our results. Another extracted factor, labelled Inefficiency, is broadly similar to Kovacs’s Ineffectiveness, but does not seem to have a counterpart in Huang and Dong’s findings. In our study, this factor includes three of the four originally proposed symptoms. Kovacs also defined ineffectiveness by the presence of self-criticism, which, in our structure, belongs to the General Symptoms factor. Inefficiency, in our results, is more directly related to the school environment, which can be explained by the age characteristics of our sample (12–16 years). In this developmental period, the school environment plays a key role in adolescents’ lives, when the gradual increase in academic demands encounters an intrapsychic upheaval associated with early adolescent development [50]. The factor labelled Social Anhedonia closely corresponds to Lack of Personal and Social Interest and Loneliness identified in Huang and Dong’s meta-analysis. In contrast to their study, in our structure, the loneliness symptom (together with suicidal ideation symptom) was assigned to the General Symptoms factor. As with the Inefficiency factor, the factor Social Anhedonia seems to be more specific than its counterpart in the meta-analysis, which can be ascribed to the extraction of the broad General Symptoms factor. Kovacs also proposed a dimension referred to as Anhedonia, but this dimension is defined more broadly to encompass, apart from social aspects, symptoms like fatigue, reduced appetite, or sleep problems, which were subsumed within the General Symptoms factor in our study. In general, the structure of CDI identified in our study is fundamentally different from originally proposed structure and also from meta-analytically derived four-factor structure suitable for non-English versions of CDI. This discrepancy can be explained by simultaneous effect of cultural and possibly language characteristics. Bonicatto et al. [26] proposed a procedure how to determine the effect of culture over and above language, which is based on comparison of instrument’s factorial structure in different countries sharing the same language, but since the Czech language is limited purely to the Czech Republic, this procedure is not applicable in our settings. However, future research could focus on differentiating sources of dissimilarities, for example by using samples consisting of bilingual individuals.

Postulating a hierarchical factor model further allowed us to speculate about the significance of the individual factors and the nature of their mutual relationships. Considering the strength of the relationships between the second-order General Depression factor and the first-order factors, it appears that General Symptoms and Negative Self-Concept might constitute core symptoms of depression, whereas Inefficiency and Social Anhedonia might be affected by additional variables unrelated to depression.

In the present study, we examined potential gender differences in the psychometric properties of the CDI, employing a multi-group CFA approach on the one hand, and an alternative approach based on the IRT on the other. Results obtained with both procedures were essentially consistent. Multi-group CFA yielded support for full measurement invariance–that is, factor loadings, item thresholds, and item uniqueness all showed a sufficient degree of equivalence in boys and girls. Similarly, an IRT DIF analysis revealed only three items functioning differentially in the two groups, and for two of these, DIF could be considered marginal. These results partly support previous findings by Gomez and Vance [25], who employed similar procedures and reported measurement invariance across genders as well, but for a younger sample of respondents (late childhood and early adolescence). On the other hand, Van Beek et al. [33] identified strong measurement bias with a large sample of children and adolescents (aged 8 to 17). In their study, about half of the CDI items showed DIF due to gender, which is in stark contrast with our findings. The only overlap between our results and the results of Van Beek et al. concerns item 10 (tearfulness), which, in both studies, was more indicative for boys when compared to girls. To quantify the potential influence of DIF items on CDI scoring, we computed the correlation between the adjusted and unadjusted overall score. Since the correlation was found to be close to 1.000, we can conclude that the three DIF-identified items did not have any clinically or practically relevant impact on the overall score.

Because the assumption of strict measurement invariance was not violated in our data, we were also able to assess latent mean differences across the two groups. The comparisons provided further evidence for substantial gender differences in depression levels in middle adolescence that cannot be viewed as mere artifacts of measurement. The greatest differences were identified in emotional and somatic symptoms of depression (which were at the core of the General Symptoms factor), but also in symptoms related to negative self-concept. Gender differences in depression were recently examined in an influential meta-analytic study [51] performed with national representative samples, which provided strong evidence for gender differences emerging as early as middle adolescence and peaking (d = 0.47) towards the end of middle adolescence. Results of this large-scale study suggest that effect sizes for gender differences in adolescent depression tend to be moderate, which is completely in line with our findings.

It is important to note that our results might have been affected by several limitations. Although our study combined exploratory and confirmatory approaches to assess the internal structure of the CDI, a replication with an independent sample is needed before our findings can be generalized to the population of Czech adolescents. Also, when interpreting our results, it is necessary to consider the relatively narrow age range of our research sample. Since the nature of the turbulent changes in emotional and psychological experience in general varies across different stages of adolescence, our results might not apply to this developmental stage in its entirety but might be only applicable for middle adolescence.

Despite the above limitations, we believe that our study constitutes a valuable contribution to the understanding of the internal structure of the CDI, especially in terms of its cross-cultural uniqueness. Our findings also support the idea that gender differences in depression can be found as early as in middle adolescence, and that these differences cannot be fully attributed to the psychometric properties of the instrument.

## Supporting information

S1 Data (SAV)
